# Relationship of visfatin with obesity and osteoporosis in patients with inflammatory bowel disease: a narrative review

**DOI:** 10.3389/fimmu.2025.1533955

**Published:** 2025-03-18

**Authors:** Alicja Ewa Ratajczak-Pawłowska, Aleksandra Szymczak-Tomczak, Szymon Hryhorowicz, Agnieszka Zawada, Kinga Skoracka, Anna Maria Rychter, Marzena Skrzypczak-Zielińska, Ryszard Słomski, Agnieszka Dobrowolska, Iwona Krela-Kaźmierczak

**Affiliations:** ^1^ Department of Gastroenterology, Dietetics and Internal Diseases, Poznan University of Medical Sciences, Poznan, Poland; ^2^ Laboratory of Nutrigenetics, Department of Gastroenterology, Dietetics and Internal Diseases, Poznan University of Medical Sciences, Poznan, Poland; ^3^ Institute of Human Genetics, Polish Academy of Sciences Poznan, Poznan, Poland; ^4^ Doctoral School, Poznan University of Medical Sciences, Poznan, Poland; ^5^ Institute of Medical Sciences, College of Social and Media Culture in Torun, Torun, Poland; ^6^ Laboratory of Molecular Genetics, Poznan, Poland

**Keywords:** obesity, osteopenia/osteoporosis, inflammatory bowel disease, bone mineral density, visfatin, Crohn disease, ulcerative colitis

## Abstract

**Background:**

Inflammatory bowel disease (IBD) is an increasingly prevalent condition in developed countries. Alongside the growing number of patients, there is a rising incidence of disease-related complications, including osteoporosis. While well-established risk factors for low bone mineral density in IBD—such as low body mass or steroid therapy—are widely recognized, other contributing factors warrant further investigation. One such factor is visfatin, a proinflammatory adipokine encoded by the *NAMPT* gene.

**Objectives:**

This review aimed to explore the association between visfatin level, bone health, and obesity among patients with inflammatory bowel disease.

**Key findings:**

Although visfatin is primarily associated with metabolic syndrome, it may also influence bone mineral density by affecting osteoblast and osteoclast differentiation and function. Additionally, some studies have identified a correlation between visfatin levels and bone mineral density. A deeper understanding of visfatin’s role in osteoporosis development may contribute to the identification of novel therapeutic strategies. Therefore, lower bone mineral density in inflammatory bowel disease may be associated with obesity and visfatin levels. However, visfatin concentrations depend on many factors, including genetics, immunology, and nutritional factors, which may affect visfatin levels.

**Implications:**

Current research highlights visfatin as both a potential biomarker and a therapeutic target for osteoporosis treatment. Nevertheless, limited studies have specifically examined the relationship between visfatin and bone mineral density in IBD. Further research is required to clarify this association and to explore how variations in visfatin levels impact bone density in IBD patients.

## 1 Introduction

Visfatin [also referred to as nicotinamide phosphoribosyltransferase (NAMPT) and pre-B-cell colony-enhancing factor (PBEF)] is an insulin-mimetic adipokine released by visceral adipose tissue. Notably, visfatin levels increase in type 2 diabetes mellitus regardless of fat distribution. While this adipokine is primarily associated with cardiovascular risk and metabolic syndrome, it also exhibits various physiological functions, including effects on cell metabolism, immunomodulation, and inflammation (1–3). Although visfatin has been primarily studied in obese patients and its consequences, some studies also link it to bone health and osteoporosis risk. Given the increasing number of individuals diagnosed with osteoporosis, there is a growing need for new therapeutic and treatment strategies for these patients.

Data about the impact of visfatin on bone mineral density (BMD) remain inconclusive. Visfatin influences bone marrow cells and osteoblasts (4). Among patients with acromegaly, visfatin correlated negatively with BMD (5). Also, in inflammatory bowel disease (IBD), the level of visfatin was associated with a risk of osteoporosis (6). However, no significant correlation between visfatin and BMD was observed in women undergoing treatment for primary osteoporosis (7). A study conducted among Chinese men and postmenopausal women also failed to identify visfatin as an independent predictor of BMD (8, 9).

IBD, including Crohn’s disease (CD) and ulcerative colitis (UC), comprises chronic inflammatory disorders that lead to multiple complications. IBD affects a growing number of individuals worldwide, with the highest prevalence reported in Europe and North America, affecting over 2 million and 1.5 million individuals, respectively (10). One of the most common complications of IBD is reduced BMD, which covers osteopenia and osteoporosis. The etiopathogenesis of bone disorders is multifactorial, with contributing factors including chronic inflammation, steroid therapy, low body mass index (BMI), or poor nutritional status (11). However, anti-TNF therapy may be beneficial for bone health (12).

Patients with IBD often present low body mass, which may lead to a decreased BMD (13). The prevalence of low BMD is highly variable and depends on factors such as population, location, or design. Osteopenia and osteoporosis affect approximately 22%–77% and 17%–41%, respectively (14). On the other hand, some IBD patients present increased fat mass, which also affects the bone negatively (15–17). Although malnutrition and underweight are common in IBD, an increasing proportion of patients are overweight (approximately 20%–40%) and obese (approximately 15%–40%) (18). It is also vital to notice that obesity may increase the risk of IBD development through dysbiosis, mucosal barrier dysfunction, or inflammation (19). In fact, fat tissue produces many adipocytes presenting pro- or anti-inflammatory action (20). One of the proinflammatory cytokines is visfatin.

According to a meta-analysis, overweight and obesity may have a protective effect against osteoporosis. However, this meta-analysis primarily focused on the association between obesity and osteoporosis, without addressing osteopenia (21). Several potential mechanisms explain how fat mass might influence BMD. First, adipocytes may affect osteoblast and osteoclast activity. Second, obesity increases insulin resistance, leading to higher insulin levels. Hyperinsulinemia, in turn, elevates free sex hormone levels by decreasing sex hormone-binding globulin (SHBG) release, which influences osteoblast and osteoclast function (21).

**Figure 1:**
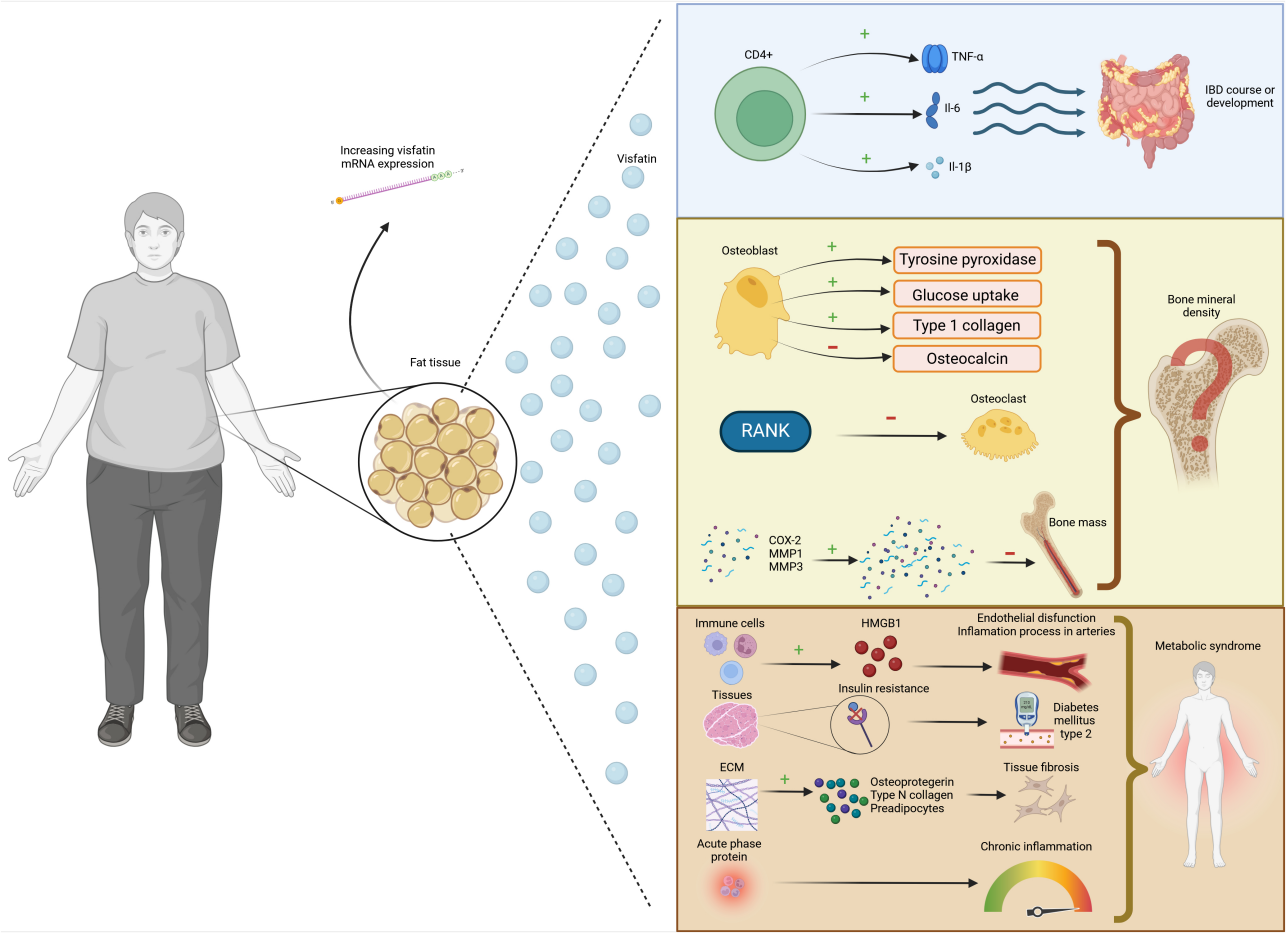
The association between visfatin, inflammatory bowel disease and osteoporosis.

For these reasons, the relationship between IBD and bone remains a topic of significant interest but is still poorly understood. This study examines the association between obesity and lower BMD and visfatin in IBD, including genetics and nutritional factors, which may affect the visfatin level. The impact of visfatin on disease risk is illustrated in **Figure 1**.

## 2 Characteristics of visfatin—genetics and immunology

**Figure 2:**
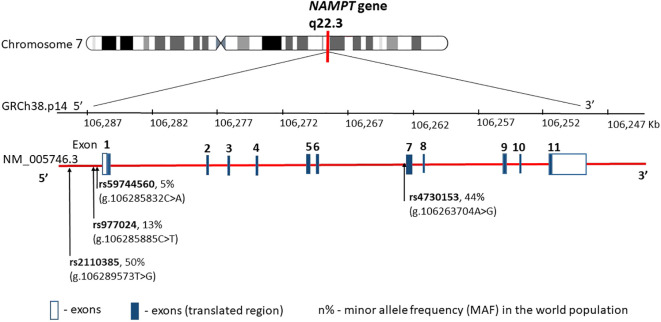
NAMPT gene structure, chromosome location, and obesity main variants distribution. rs—number of the reference sequence in the National Center of Biotechnological Information database.

The human visfatin gene, currently known as nicotinamide phosphoribosyltransferase (*NAMPT*), consists of 11 exons and is located on the 7q22.3 encoding a polypeptide of 491 amino acids (22) (**Figure 2**).

It turns out that the expression of visfatin mRNA increases during the development of obesity, and its plasma level strongly correlates with the amount of visceral fat (1, 17, 20, 22, 23).

Among the 52 single nucleotide polymorphisms (SNPs) of visfatin described to date, some are associated with determinants of obesity and glucose/lipid metabolism (24–28). The SNPs most commonly described in the literature in this context are SNPs rs4730153 and G-948T (rs59744560), where the G-948T alteration is associated with glucose/lipid metabolism and obesity-related conditions. A German group tested three representative single nucleotide polymorphisms (rs9770242, -948G>T, rs4730153) in 731 school-aged children and an independent cohort of 167 obese children showing no association of any of the three polymorphisms or their haplotypes with body mass index (BMI), waist-to-hip ratio, or parameters of glucose, insulin, or lipid metabolism (29). In contrast, an Iranian group concluded that visfatin G-948T polymorphism was correlated with obesity, total cholesterol, and LDL-C levels in the Iranian population (30). *NAMPT* genetic variants have also been shown to be associated with obesity and to affect the levels of visfatin/NAMPT in severely obese children (24). However, none of the previous studies considered the interactions between *NAMPT* polymorphisms. Therefore, combinations of genetic variants may be much more important in the pathogenesis of obesity.

Visfatin expressed predominantly in visceral fat is a 52–55-kDa proinflammatory adipokine (23, 31). It was first described by Samal (22) and colleagues in 1994 as pre-B-cell colony-enhancing factor 1 (*PBEF1*) and then in 2004 by Fukuhara et al. (23) as an adipokine with insulin-mimetic effects. The effects of visfatin on adipogenesis and glucose metabolism are of particular interest with regard to its role in obesity and diabetes. However, studies on the relationship between visfatin and obesity have yielded conflicting results. Visfatin is recognized as a proinflammatory cytokine associated with cellular metabolism (25, 32), immunomodulation (26, 32), and inflammation (27, 28). It is found in various immune cells (not B lymphocytes), inhibiting macrophage apoptosis (4). In addition, extracellular PBEF promotes proinflammatory cytokines such as tumor necrosis factor (TNF)-α, interleukin (IL)-1, IL-16, transforming growth factor (TGF)-β1, and the chemokine receptor CCR3. It also increases the production of IL-6, TNF-α, and IL-1 in CD14^+^ monocytes, macrophages, and dendritic cells, improves the function of T lymphocytes, and is essential for the development of B and T lymphocytes (32).

Adipocytes and infiltrating macrophages secrete visfatin in adipose tissue but are also present in hepatocytes, skeletal muscle cells (33, 34), marrow, macrophages, liver (35), immune cells (36), brain cells (37), and glomeruli (38). This protein has insulin-mimetic properties and directly binds to the insulin receptor at a site other than the insulin, leading to increased glucose uptake in muscles and adipose tissue. It inhibits hepatic gluconeogenesis *in vitro* and *in vivo*, thus showing hypoglycemic effects and playing a positive role in undermining insulin resistance.

Visfatin is expressed by macrophages infiltrating adipose tissue (39). This prompts the hypothesis that visfatin may be involved in some complications of obesity, such as metabolic syndrome and/or type 2 diabetes mellitus (T2DM).

## 3 Visfatin and obesity

Visfatin is a proinflammatory protein from the adipokine group, whose association with metabolic syndrome and obesity has been the subject of many scientific studies. Chang et al. (40) demonstrated a significant increase in visfatin levels in overweight or obese patients and those with type 2 diabetes. Additionally, higher visfatin levels were observed in individuals diagnosed with metabolic syndrome and cardiovascular disease. It is likely to play an important role in the early stages of disease progression. Increased visfatin production has been linked to high mobility group box 1 (HMGB1), which enhances inflammatory processes in the arteries and contributes to endothelial dysfunction (41, 42). Moreover, visfatin upregulates extracellular matrix (ECM) proteins, including osteopontin, type VI collagen, and preadipocytes, which can exacerbate tissue fibrosis (43). A study by Ugur et al. showed a direct relationship between the amount of visfatin and other adipokines and body weight, diastolic blood pressure, and the amount of HDL cholesterol (44). Similarly, a study by Jurdan et al. found higher baseline fasting visfatin values in overweight and obese subjects than in controls (45).

However, some studies suggest that increased visfatin levels are more strongly associated with type 2 diabetes than with obesity alone (46). In the study of Takebayashi, visfatin levels were significantly elevated in patients with type 2 but remained comparable in obese subjects without diabetes and lean controls. Moreover, a pathophysiological mechanism of the association between elevated visfatin levels and the development of gestational diabetes in obese women has also been demonstrated (47).

Visfatin secreted by visceral fat cells has anabolic effects similar to insulin (48). It binds to the insulin receptor with an affinity comparable to insulin, albeit at a different binding site. Visfatin also directly increases insulin resistance in peripheral tissues, leading to the development of prediabetes and type 2 diabetes (49). However, some studies have found no significant association between circulating visfatin levels and insulin sensitivity or glucose tolerance (50). The relationship between elevated visfatin levels and obesity-related fatty liver disease remains unclear. In a study by Chen, significantly higher visfatin levels were observed in patients with MAFLD from the Middle East and in obese individuals compared to controls. This finding underscores the need for further research on the topic (51). Conversely, a meta-analysis by Ismaiel found no association between visfatin levels and NAFLD, simple steatosis, or liver fibrosis (52).

Visfatin also exhibits proinflammatory properties by releasing acute-phase proteins and proinflammatory cytokines (e.g., IL-6), significantly affecting metabolic disease development (53). Additionally, it increases TNF-α levels via Sirt6-mediated pathways (54). Its atherogenic effects are related to the activation of MMPs and the promotion of lipoprotein oxidation by phospholipases, which affects atherosclerotic plaque destabilization, which is associated with an increased risk of acute cardiovascular diseases. Furthermore, visfatin induces the proliferation of vascular smooth muscle cells and fibroblasts, affecting cardiac remodeling and leading to myocardial fibrosis. This fibrosis impairs the contractile function of the heart muscle, resulting in cardiological complications in obese individuals (55). The role of visfatin in macrophage polarization is also noteworthy. A study involving 88 tissue samples revealed that visceral adipose tissue macrophages exhibited higher visfatin levels than mature adipocytes. Visfatin’s association with obesity is also significant in terms of cancer incidence, as obesity has been linked to a higher risk of malignancies (56). It also has implications for patients with inflammatory bowel diseases. Its association with colorectal cancer is well-documented (57), and this effect may be exacerbated in individuals with IBD, where the risk of colorectal cancer is even greater. This effect may be compounded in people with inflammatory bowel disease, in whom this risk is even greater. Assessing visfatin levels in at-risk individuals may have clinical implications. The carcinogenic mechanism of visfatin is related to its role in promoting the survival of cancer cells. Specifically, intestinal cancer cells express the chemokine receptors CXCR4 and CXCR7, which bind to stromal cell-derived factor 1 (SDF-1), and their production is upregulated in response to elevated visfatin levels (58, 59). In addition, visfatin increases the activity of antioxidant enzymes [superoxide dismutase (SOD), catalase (CAT), and glutathione peroxidase] (60), which induces protection of cancer cells from cytotoxic damage by reactive oxygen species (61). In the context of obesity, visfatin levels are also associated with an increased risk of osteoporosis and osteoporotic fractures (62). Moreover, its insulin-mimetic properties have been demonstrated to enhance glucose uptake in human osteoblasts (63). The most relevant studies on the relationship between obesity and visfatin levels are summarized in **Table 1**.

**Table 1:** Summary of the most important human studies on the association between obesity and visfatin level.

Studies	Methodology	Outcome
Chang et al. (40)	Meta-analysis of 46 studies	Plasma visfatin is significantly increased in overweight or obese patients with diabetes mellitus type 2, metabolic syndrome, and cardiovascular disease
Ugur K. et al. (44)	Comparing visfatin concentration between: • Patients with obesity metabolic syndrome • Patients without obesity, but with metabolic syndrome • Control group	• Negative correlation between visfatin and BMI in the metabolic syndrome group • No significant differences in visfatin level between groups • Negative correlation of visfatin with waist circumference in a group with obesity and metabolic syndrome and the group with metabolic syndrome
Jurdana M. et al. (45)	Comparison of anthropometric and laboratory parameters, including visfatin levels, among 48 normal-weight adults and 48 overweight adults	• Visfatin level is higher in overweight than in normal-weight adults • Positive correlation between visfatin and triglycerides, CRP, and TNF-α • Physical fitness is correlated negatively with visfatin
Chen S. et al. (51)	Meta-analysis of seven studies comparing visfatin levels between patients with metabolic fatty liver disease and a control group	No significant differences in visfatin levels between patients with metabolic fatty liver disease and the control group

## 4 Visfatin and diet

The study by Perez-Echarriet al. suggests that in animal models, overfeeding with a saturated fatty acid-rich diet (so-called cafeteria diet) can impair visfatin gene transcription in white adipose tissue (64). Rats fed a cafeteria diet had significantly decreased visfatin mRNA concentrations in visceral fat tissue. Interestingly, administration of 1 g/kg of eicosapentaenoic acid ethyl ester prevented the decrease of visfatin gene expression. Also, other fatty acids—oleic and palmitic acids—can also regulate visfatin gene expression. In the *in-vitro* study of Wen et al., oleate and palmitate significantly and dose-dependently downregulated visfatin mRNA concentrations in adipocytes and preadipocytes—maximally by 47% and 45% for palmitic acid and oleic acid, respectively (65).

On the other hand, a diet rich in sugar-sweetened beverages and low in fruits did not influence visfatin concentrations among individuals with increased body weight (66). However, fish consumption significantly and positively correlated with visfatin concentrations (0.063). In another study, although hypertensive subjects had significantly higher plasma visfatin concentrations, a sodium-restricted diet did not influence visfatin levels among individuals with visceral obesity (67).

Considering the proinflammatory effect of visfatin, it is also worth quoting the results of a study conducted by Hernando-Retondo et al., who compared an intervention with a caloric-restrictive MedDiet plus physical activity promotion versus a non-restrictive MedDiet and then compared the results to a control group. At 6- and 12-month follow-ups, the authors assessed the impact on satiety-related hormones, lipid and glucose metabolism, and inflammation in adults with metabolic syndrome. A reduction in visfatin levels was observed in both groups with an initial decrease at 6 months followed by steady maintenance at 12 months (68). Similar results were obtained in another study, where a hypocaloric diet followed by a 3-month weight reduction significantly decreased visfatin concentrations among obese individuals (112.14 ± 70.2 vs. 99.4 ± 58.1 ng/mL, *p* < 0.05) (69). On the other hand, in the study of De Luis et al., a 2-month significant weight reduction (113.1 ± 18.9 vs. 108.5 ± 18.1 kg, *p* = 0.001) did not influence visfatin concentrations among obese individuals (43.5 ± 30.8 vs. 47.1 ± 38.1 ng/mL) (70).

Dinu et al., in a randomized, open, crossover trial, observed a significant decrease of visfatin only after 3 months with vegetarian diet (VD) intervention and not after the Mediterranean diet (MD) (71). On the other hand, the study conducted by Ambroszkiewicz et al. did not show any influence of consuming a VD on the serum levels of visfatin, and there were no statistically significant differences in the median values of visfatin serum levels between vegetarians and omnivores (72). Several other studies have also shown the lack of a relationship between hypocaloric MD and plasma concentration of visfatin (73–75).

A very interesting nutrigenomics study was conducted by Khorrami-Nezhad et al. in a group of 336 obese individuals. The authors searched for the interaction between visfatin genotypes and dietary fat intake, with regard to BMD. Participants were divided into three groups according to the presented visfatin genotype. It turned out that women with the TT genotype had higher lumbar BMD, whereas those with the GT genotype had higher hip BMD. The amount of fat in the diet (<30% vs. >30% of DCI) did not play a crucial role in BMD determination, but the type of fatty acids had an impact on BMD.

However, apart from the study conducted by Khorrami-Nezhad, no other studies have been conducted in the context of osteoporosis, so we believe this is a direction that should be developed. In the context of obesity-related dietary factors, more studies are also needed in order to confirm *in-vitro* and animal studies among the human population and to provide a basis for more precise nutritional guidelines.

## 5 Visfatin in IBD

There is a relationship between obesity and the risk of IBD. White adipose tissue contains adipocytes, producing adipocytokines, which may affect the immune system and lead to inflammatory disease development, including IBD (76). One such adipokine is visfatin, which may activate inflammatory pathways by stimulating the production of proinflammatory cytokines such as TNF-α, IL-6, and IL-1β by CD14(+) monocytes (77, 78). These mechanisms might play a role in the onset of IBD or influence its progression.

Saadoun et al. reported that newly diagnosed IBD patients presented higher concentrations of visfatin than healthy controls. Additionally, serum visfatin correlated negatively with serum albumin and positively with CRP and ESR in UC patients. A positive correlation between visfatin concentration and fecal calprotectin was observed in both CD and UC.

Differences in visfatin levels were also noted depending on disease severity. According to the Montreal classification, patients with extensive UC (E3) had higher vastatin levels when compared to UC patients with proctitis (E1) and left-side colitis (E2). Additionally, CD patients with structuring phenotype (B2) presented higher concentrations of vastatin than patients with non-structuring and non-penetrating CD phenotype. However, it is important to note that this was a single-center study with a small sample size (fewer than 100 IBD patients) (79). Visfatin levels tend to be higher in CD than in UC patients. The authors found no correlation between visfatin and age, BMI, or CRP (6). However, a Mexican study showed a lack of differences in visfatin levels between IBD and healthy groups (80).

According to Dogan et al., patients with active UC presented a higher concentration of visfatin than UC patients in post-treatment remission as well as healthy subjects (81). Waluga et al. also reported that serum visfatin concentration decreased after 3 months of steroid and/or azathioprine treatment in CD patients but not in the UC group (82). Moreover, the amount of visfatin per milligram of colon biopsy protein was higher in IBD patients than in healthy control, and visfatin levels in CD pediatric biopsies correlated to the pediatric Crohn’s disease activity index (PCDAI) score (83).

Visfatin levels correlated with the presence of osteoporosis in IBD (6). PBEF/NAMPT/visfatin correlated negatively with BMD and positively with disease activity in IBD (84). Moreover, visfatin levels exhibited an inverse correlation with vitamin D levels among UC patients with vitamin D insufficiency. However, this study included only patients in remission, and the authors did not analyze inflammatory factors, which may also have influenced the results (85). Although limited studies specifically address the impact of visfatin on BMD in IBD, there is research examining the association between BMD and body composition, which may be linked to adipocyte activity. Hip BMD has been found to correlate negatively with fat mass percentage. Additionally, the trabecular bone score is negatively associated with visceral fat mass as a percentage of total fat (86). On the other hand, in a 24-month observational study, the obesity rate increased, and lean muscle mass decreased, while the frequency of osteopenia remained unchanged (17).

The association between visfatin, bone mineral density, and other analyzed parameters represents only a snapshot in time, highlighting the need for further longitudinal studies.

## 6 Visfatin and osteoporosis

Osteoporosis is a generalized skeletal disease characterized by progressive loss of bone mineral density, altered bone spatial structure, and increased susceptibility to fracture (87). Risk factors for osteoporosis include older age, female gender, onset of menopause, low body weight, immobility, low physical activity, hypogonadism, low calcium intake, vitamin D deficiency, smoking and alcohol abuse, and use of certain medications, particularly glucocorticosteroids (88). Osteoporosis, according to the World Health Organization (WHO) guidelines, is diagnosed by measuring BMD of the hip and spine using dual-energy X-ray absorptiometry (DEXA), based on the T score expressed as the number of standard deviations, with peak bone mass as the reference point (88).

Bone tissue undergoes constant remodeling involving the main bone cells—osteoblasts and osteoclasts. Osteoporosis is believed to result from an imbalance between bone formation and resorption, which can lead to increased bone turnover (89). The molecular mechanisms regulating bone mineral density loss are complex and involve numerous factors. It has been suggested that adipokines such as visfatin, resistin, and leptin may influence bone homeostasis cells either directly or indirectly (90, 91).

The study by Tariq et al. which included 72 postmenopausal women with normal BMI, 72 postmenopausal women with osteopenia, and 100 postmenopausal women with osteoporosis showed that serum visfatin levels were statistically significantly lower in women with osteopenia and postmenopausal osteoporosis compared to healthy women. In addition, visfatin was also found to be an independent positive predictor of bone mineral density at the lumbar spine and femoral neck (92). Similar observations were made by Siviero-Miachon et al. In this study, the authors recruited 56 patients 15–24 years old, who survived acute lymphocytic leukemia; in this group of patients, both lumbar spine BMD and total BMD positively correlated with visfatin levels (93). Also, Iacobellis et al. enrolled in their study 72 patients with metabolic syndrome (25 men and 47 women) with a mean age of 58.14 ± 11 years and showed that plasma visfatin levels were positively correlated with L2–L4 BMD in men (94).

However, the studies of Peng et al. and Gruodytė et al. found no association between visfatin levels and BMD in Chinese men aged 20–80 and young female athletes (8, 95). Biver et al., in a meta-analysis of 59 studies, also found no association between visfatin and BMD. The researchers emphasized that the inconsistent relationship between adipokines and bone mineral density may be disrupted by body composition parameters, particularly fat mass (96). The differences in the discussed studies can be caused by different populations, sex or age of the participants, and disease occurrence. Additionally, the above studies presented relatively few groups.

Visfatin has a stimulatory effect on osteoblastogenesis and may also alter osteoblast differentiation and function (93, 97). Visfatin can stimulate osteoblast growth, increase type 1 collagen expression, and stimulate mineralization (98). Xie et al. showed that visfatin induces tyrosine phosphorylation within the insulin receptor, which was identified in osteoblasts. In cultured human osteoblast-like cells, visfatin increased glucose uptake and proliferation and growth of type 1 collagen. Visfatin also inhibited osteocalcin secretion from human osteoblast-like cells (63). Inhibition of visfatin in mouse bone marrow mesenchymal cells (BM-MSCs) or visfatin knockout in mice led to reduced osteoblastogenesis, and it has been shown that lowering serum levels of visfatin also reduced alkaline phosphatase activity and inhibited matrix mineralization, resulting in decreased bone formation (97). The mechanism underlying this visfatin-promoting osteoblast differentiation can be partially explained by an epigenetic process involving the modification of H3-Lys9 acetylation (99).

Visfatin may also inhibit osteoclastogenesis. It has been shown to suppress the production of osteoclast differentiation and activity markers, including RANK, cathepsin-K, and the nuclear factor of activated T cells c1 (NFATc1) (100). This finding aligns with another *in-vitro* study on bone marrow-derived macrophages, in which visfatin inhibited RANKL-mediated activation by preventing the phosphorylation of several key signal transduction proteins involved in osteoclastogenesis. Notably, the activity of mature osteoclasts remained unchanged (101).

However, the effect of visfatin on bone cells may also be associated with promoting inflammatory responses in these cells and thus may have a catabolic effect on bone tissue (102). The link between visfatin and increased inflammation and the consequent inhibition of bone metabolism was confirmed by animal models of inflammation. In these models, visfatin promoted bone loss and other catabolic and inflammatory responses by upregulating COX-2, MMP1, and MMP3. In contrast, inhibition of visfatin by FK866, a highly specific non-competitive inhibitor, led to a reduction in proinflammatory factors (Il6, Il8/Kc, and Mcp1) in mouse osteoblasts (103). The most important studies on the association between obesity and visfatin level are shown in **Table 2**.

**Table 2:** Summary of the most important human studies on the association between bone and visfatin level.

Studies	Methodology	Outcome
Tsiklauri L. et al. (90)	Assessment of gene expression of visfatin bone marker genes	Increased visfatin levels in the group with femoral neck fracture and osteoarthritis Visfatin increased matrix mineralization and reduced collagen type I expression
Tariq S. et al. (92)	Comparing postmenopausal women with normal bone mineral density, osteopenia, and osteoporosis 50–70 years of age	Women with osteopenia or osteoporosis presented lower visfatin levels than the control group Visfatin level was a positive predictor of low bone mineral density
Siviero-Miachon A. A. et al. (93)	The cross-sectional study included 56 patients who received cranial radiotherapy aged 15–24 years	Lumbar spine (L1–L4) and total body correlated positively with visfatin level
Iacobellis G. et al. (94)	Assessment of bone mineral density and visfatin level among: • 72 patients with metabolic syndrome • 40 control subjects	Visfatin correlated positively with bone mineral density of L2–L4 in the male group
Gruodytė R. et al. (95)	Assessment of visfatin levels, bone mineral density, and bone mineral content of femoral neck and lumbar spine (L2–L4) in 170 healthy female athletes and sedentary controls (*n* = 33)	There is no association between visfatin levels and bone mineral density or bone mineral content in female athletes and the control group

Understanding the mechanisms involved in the differentiation and homeostasis of bone tissue cells is crucial for researching bone mineral density disorders. These findings could contribute to the identification of markers to prevent the onset of disease, monitor its progression, and develop new therapies. To date, research on visfatin has shown promise as both a marker and a potential therapeutic target for treating osteoporosis. It is worth noting, however, that the complex interaction between adipogenic factors such as visfatin and osteogenic factors, as well as the production of these molecules by peripheral adipose tissue and bone marrow adipocytes, complicates the assessment of visfatin’s clinical utility. This challenge underscores the need for further research (100). Ling et al. showed that visfatin plays a critical role in osteoblast differentiation and stimulates osteogenesis by epigenetically regulating Runx2 expression, demonstrating that visfatin may be an important therapeutic target in senile osteoporosis (99). Currently, there are a few visfatin inhibitors; three of them, CHS828, FK866, and KPT-9274, have been examined in clinical studies. Probably, these will be the main research goals in the coming years. Although the final results of these studies are not yet available, initial reports indicate potential side effects such as anemia, thrombocytopenia, and hypoalbuminemia.

Ok et al. presented that neutralizing visfatin activity through blocking antibodies inhibits osteoclastogenesis induced by RANKL. This emphasizes the role of visfatin in osteoclastogenesis induced by RANKL *in vitro* and draws attention to the RANKL/visfatin signaling pathway as a potential aim of therapy in diseases associated with loss of body mass (104).

Further *in-vitro* and *in-vivo* studies are needed to clearly define the role of visfatin in diagnosing and treating bone mineral density disorders. It is vital to notice that *in-vivo* studies are crucial for the assessment of the safety, effectiveness, and potential interaction of visfatin inhibitors.

## 7 Summary and conclusion

Visfatin is a ubiquitous adipokine with catabolic and proinflammatory properties. As a cytokine, it exerts immunomodulatory effects, has insulin-mimetic properties via interaction with the insulin receptor, has catalytic functions, is potentially involved in carcinogenesis, and possibly induces oxidative stress in cells. It is important to understand the complex mechanisms of action of visfatin in other diseases, especially among patients with inflammatory bowel disease and osteoporosis. Clinicians are exploring visfatin as a potential therapeutic target; however, further research is required, particularly *in-vivo* studies, to assess the safety and efficacy of visfatin inhibitors for osteoporosis treatment in IBD patients. Initial studies have reported anemia as a side effect of visfatin inhibitors, indicating that these agents may not be the optimal therapeutic option for managing low BMD in IBD.
